# Urinary concentrations of ADAM 12 from breast cancer patients pre- and post-surgery vs. cancer-free controls: a clinical study for biomarker validation

**DOI:** 10.1186/1477-5751-13-5

**Published:** 2014-04-01

**Authors:** Erin K Nyren-Erickson, Michael Bouton, Mihir Raval, Jessica Totzauer, Sanku Mallik, Neville Alberto

**Affiliations:** 1 Department of Pharmaceutical Sciences, Fargo, ND 58108-6050, USA; 2 Department of Pharmacy Practice, North Dakota State University, Fargo, ND 58108-6050, USA; 3 Essentia Health, Fargo, ND 58103, USA; 4 Roger Maris Cancer Center, Sanford Health, Fargo, ND 58122, USA

**Keywords:** A Disintegrin and Metalloproteinase, ADAM12, ADAM12-S, Breast cancer, Cancer biomarker

## Abstract

**Background:**

The ADAMs (A Disintegrin and Metalloproteinases) are a family of multi-domain, zinc-dependent metalloproteinase enzymes. ADAM 12 has been previously associated with the onset and progression of breast cancer, and elevated levels of ADAM 12 have been previously found in the urine of breast cancer patients. Aims of the current study are: **1**) establish the viability of urinary ADAM 12 as a diagnostic marker for breast cancer, and **2**) explore the effects of surgical tumor removal on the levels of urinary ADAM 12.

**Methods:**

A total of 96 patients have been recruited for this study, including 50 patients diagnosed with cancer, and 46 age-matched controls. Commercially available ELISA kits for ADAM 12 were used to quantify the presence and concentration of this enzyme in the urine from cancer patients with ductal carcinoma *in situ* (DCIS) and invasive breast cancer (IBC) both prior to any treatment and approximately two weeks following surgery, as well as from controls.

**Results:**

We find no statistically significant differences between the concentrations of ADAM 12 in the urine of breast cancer patients prior to treatment and that of their age-matched controls; however the concentration of ADAM 12, both alone and as a function of urine total protein, are significantly elevated following surgery (p < 0.0001). Patients who underwent a mastectomy have significantly higher urinary ADAM 12 concentrations than those who underwent a lumpectomy (significant at p = 0.0271).

**Conclusions:**

These findings suggest that urinary ADAM 12 may not correlate directly with the status and stage of breast cancer as previously thought; rather these increases may be a result of tissue injury and inflammation from biopsy and surgical resection. Results of this study may suggest a need for biomarkers to be evaluated carefully in the context of tissue damage.

## Background

Breast cancer is currently the second leading cause of cancer deaths among women in the United States (second only to lung cancer), and it is now estimated that in the U.S. one in eight women will be diagnosed with breast cancer during her lifetime [1]. However, if breast cancer is detected during its earlier stages, the 5-year survival rate may be as high as 93% (at stage 0; this reflects death from all causes); when detected at stage IIIB and later, 5-year survival rate drops below 50% [2], making early detection of breast cancer essential for favorable prognosis. Tumor markers currently in use in the evaluation of breast cancer include (but are not limited to) cancer antigen 15-3 [3,4] and 125 [5] (CA15-3, CA 125), carcinoembryonic antigen [3,4] (CEA), and prolactin [6]; however these show little potential for early detection [7]. Recent studies have begun exploring the potential of urinary biomarkers for breast cancer detection [8,9], and among those studied are the ADAM proteases [10], particularly ADAM 12 [11-13].

The ADAMs (A Disintegrin and Metalloproteinases) are a family of multi-domain, zinc-dependent metalloproteinase enzymes. There are currently 40 known genes for ADAMs, 21 of which are known to function in humans [14]. ADAMs are usually membrane bound (although some members of the ADAM family have secreted forms, including ADAM-9 [15], -10 [16], -12 [17] and -28 [18-20]), and their physiological roles include extracellular matrix restructuring [21-23], cell adhesion [24-26], and cell-surface protein processing [27-29]. ADAM 12, which is transcribed as both a membrane bound (ADAM 12-L) and a secreted form (ADAM 12-S), has roles in cell adhesion and matrix restructuring during cell differentiation [24-26,30], and also has regulatory functions [31] in healthy tissues. ADAM 12 has also been associated with development and progression of a number of disease states, including arthritis [32], cardiac hypertrophy [33], liver fibrogenesis [34], and various cancers, including bladder [35], lung [36], brain [37] and breast [11]. It has also been suggested that the ADAM 12 produced by the tumor cells drives the progression of breast tumors [38]. Further, a study conducted in 2012 correlates gene expression of both ADAM 12 and ADAM 17 with clinical and pathological characteristics of breast cancer [39].

One study in 2004 indicated a strong correlation between excretion of urinary ADAM 12 and breast cancer status and stage [11]. This report concluded that patients with ductal carcinoma *in situ* (DCIS), invasive breast cancer (IBC), and metastatic breast cancer had significantly higher levels of ADAM 12 present in their urine than controls (i.e. patients with “no discernible disease”) [11]. The report further concluded that only 15% of the control subjects had detectable levels of ADAM 12 present in their urine, while 82%, 86%, and 85% of patients with DCIS, IBC and metastatic disease, respectively, were positive for the presence of ADAM 12 [11]. These results strongly suggest that a urine test for ADAM 12 would prove especially useful for the diagnosis of breast cancers, stage DCIS and later.

The aims of the current study are twofold: **1**) establish the viability of urinary ADAM 12 as a diagnostic marker for breast cancer, and **2**) explore the effects of surgical tumor removal on the levels of urinary ADAM 12. We hypothesize that, as previous reports suggest that breast tumor progression is responsible for the increased ADAM 12, the urinary ADAM 12 concentrations will be significantly higher in cancer patients than controls, and that urinary ADAM 12 levels will decrease following tumor removal. Our primary objective is to establish a simple, practical test for the early detection of breast cancer. As such, we have chosen to utilize commercially available ELISA kits for urinary ADAM 12 measurements: we reason that they are a well accepted technology, which will provide reliable, reproducible results in a clinic setting.

## Methods

### Ethics review and approval

This study was conducted in compliance with the Helsinki Declaration. The protocol, informed consent form, and laboratory manuals for this study were reviewed and approved by the Sanford Health Institutional Review Board in compliance with its Federalwide Assurance (#00016819). All patients’ participation was voluntary, and all enrolled participants were given the right to refuse or exit the study at any time. Participants’ were given a unique study number; and therefore their specimens and related medical information were de-identified. Participant’s study related medical record information was protected in accordance with HIPAA regulation.

## Materials

Coomassie Blue (Bradford) Assay Kit was obtained from Thermo Scientific (Rockford, IL), and 96-well polystyrene plates for this assay were obtained from Greiner Bio-One (Monroe, NC). ADAM 12 enzyme linked immunosorbent assay (ELISA) kits obtained from R&D Systems (Minneapolis, MN). All supplies used without any further modifications.

### Patient recruitment

Study participants were screened during their visit either to the Sanford Breast Clinic and/or breast surgeon consultation visit by their treatment provider. If they wished to participate in the study, the clinical research coordinator met with them to discuss and/or complete the Informed Consent Form (ICF) document and process. After consent was obtained, the coordinator collected the pre-surgery or control urine sample, and informed those participants with breast cancer of the need to leave a second sample at a follow-up visit after their surgery. Control patients were matched for age and co-morbidities. They were selected from Sanford Medical Center Breast Clinic or other clinics. If they had benign and non interventional breast findings they were approached at their clinic visit about participation in the study and appropriately consented. Controls were consented using the same ICF document and process as breast cancer subjects.

Inclusion criteria:

• Females age 21 years of age or older

• Recent diagnosis of breast cancer

• No previous diagnosis of cancer, excepting non-melanoma skin cancer.

• Treatment naïve (i.e., no chemotherapy or radiation therapy prior to surgery for this breast cancer diagnosis)

Exclusion criteria:

• Pregnancy

• Advanced stage breast cancer disease (i.e., stage 4 cancer with multiple metastasis)

Age-matched controls were females with no positive history of breast cancer or other previous diagnosed cancers, excluding non-melanoma skin cancer. All patients were recruited for study from the Sanford Medical Breast Clinics, and were consented in accordance with institutional regulatory board guidelines. All breast cancer patients had surgery as their initial treatment. The surgeries involved either a lumpectomy or a mastectomy for local control of their cancer; and in most cases axillary sentinel lymph node dissection was included. When indicated, a level I and II axillary lymph node dissection was done as part of the same procedure based on frozen section evaluation on the sentinel lymph nodes. The decision of surgery options was made after multidisciplinary treatment planning, consultation with the patients, and followed National Cancer Cooperative Network (NCCN) guidelines.

### Urine collection and processing

Following consent, patients and controls were brought to a private area and asked to leave a urine sample. Immediately following collection, the urine was well mixed, and ten milliliters (10 mL) was aliquoted into a sterile, 10 mL screw cap test tube, and labeled with the patients de-identified information only; available information includes only patient age, stage of cancer, tumor size and co-morbidities. These samples were immediately placed upright in a -80°C freezer for storage. Recruited breast cancer patients provided two samples of urine, one just following diagnosis, and a second approximately two weeks following surgery to remove the tumor mass (all patients recruited for this study were scheduled for surgery). Controls have provided one sample only. Upon collection of 20 samples, tubes were transported to North Dakota State University on dry ice for testing.

Prior to testing, samples were thawed on ice and centrifuged at 200 rcf for 15 minutes to remove any particulates. The resulting supernatant was diluted 1:5 in one of two buffers: for the Bradford assay, 50 mM Tris at pH 8 was used, and for ELISA the calibrator diluent provided with the kit was used, as per the manufacturer’s suggestion. Preliminary data demonstrated the necessity of dilution such that the patient samples would fit within the standard curve of both the Bradford assay and the ADAM 12 ELISA.

### Bradford assay

Manufacturer’s instructions for the “micro microplate procedure” obtained with the kit were followed regarding volumes of samples, standards, and assay reagent. Bovine serum albumin was provided with the kit, and was used to produce the standard curve. The 2 mg/mL albumin standard was diluted in 50 mM Tris buffer (pH 8) to produce a standard curve ranging from zero μg/mL to 100 μg/mL. Twenty patient and/or control urine samples diluted 1:5 (see Section on Urine collection and processing) were loaded into four wells each of a 96-well standard clear bottom polystyrene plate, 150 μL per well. Standard samples were also loaded, two wells each sample, 150 μL per well. Bradford assay reagent provided was loaded into each well, 150 μL per well, and the plate was mixed on a shaker for 10 seconds, followed by incubation at room temperature for 10 minutes. Reading of plate absorbance, production of the standard curve and analysis of the samples was performed according to manufacturer’s instructions.

### ELISA

Twenty patient and/or control urine samples diluted 1:5 (see section on urine collection and processing) were loaded into four wells each of the provided 96-well plate of a commercially available ELISA kit. Standard samples were also loaded, two wells each. Manufacturer’s instructions were followed for production of standard curve and analysis of samples. The antibodies provided with this kit consist of monoclonal antibodies specific for ADAM 12.

### Statistical analysis

Groups were compared using nonparametric Mann-Whitney test (α = 0.05). Analysis was performed using Minitab (v. 16.1.1).

## Results

**Figure 1:**
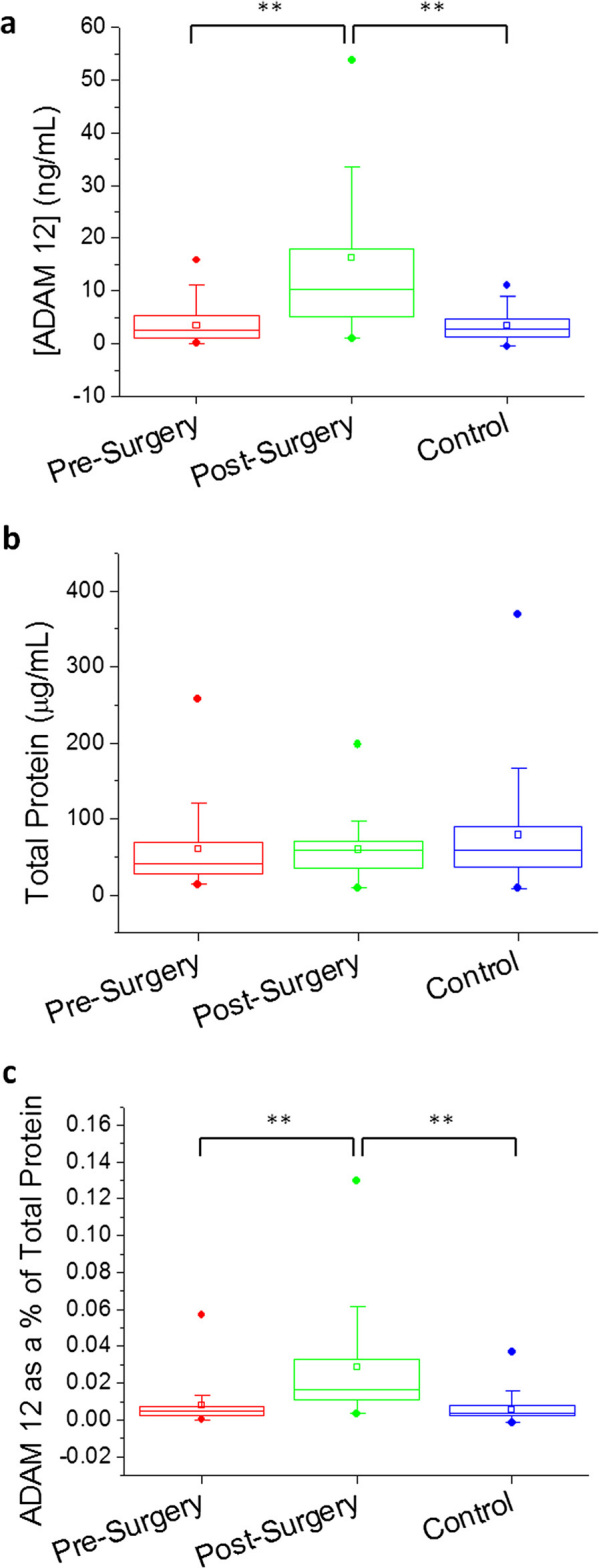
**Boxplots showing urinary ADAM 12 levels in patient groups (pre- and post-surgery) vs. control group; concentration of ADAM 12 denoted in ng/mL (a), total protein concentration (b), and ADAM 12 as a% of total protein (c) are shown for each respective group.** Circles above and below the box denote 99% and 1%, respectively; vertical bars denote 10-90%; the box denotes 25-75%; the small square denotes the mean; and the horizontal bar denotes the median. ** p < 0.0001.

A total of 50 patients with the diagnosis of breast cancer and 46 age matched control patents were recruited into the study. Based on the data collected, no significant differences exist between the urinary ADAM 12 concentrations of the control patients and the cancer patients prior to their surgery. The urinary concentration of ADAM 12 increased significantly following patient surgery (p < 0.0001), both in ng/mL and as a function of total urine protein. Results are summarized in Table 1 and Figure 1 below.

**Table 1:** Urinary ADAM 12 of cancer patient group vs. control group

	**Cancer group: pre-surgery**	**Cancer group: post-surgery**	**Control group**
	**(n = 50)**	**(n = 49)**	**(n = 46)**
Age	Mean: 60.9 ± 13.0	Mean: 60.9 ± 13.0	Mean: 60.1 ± 12.4
	Median: 60	Median: 60	Median: 60
[ADAM 12] (ng/mL)	Mean: 3.36 ± 3.04	Mean: 16.3 ± 15.6	Mean: 3.52 ± 3.17
	Median: 2.48	Median: 10.3	Median: 2.77
Total protein (μg/mL)	Mean: 60.7 ± 53.6	Mean: 59.7 ± 32.9	Mean: 78.6 ± 65.6
	Median: 42.0	Median: 59.0	Median: 58.7
ADAM 12 as % of total protein	Mean: 0.008 ± 0.01	Mean: 0.03 ± 0.03	Mean: 0.006 ± 0.006
	Median: 0.005	Median: 0.017	Median: 0.004

Cancer group consists of DCIS (n = 15) and IBC (n = 35) diagnoses.

**Figure 2:**
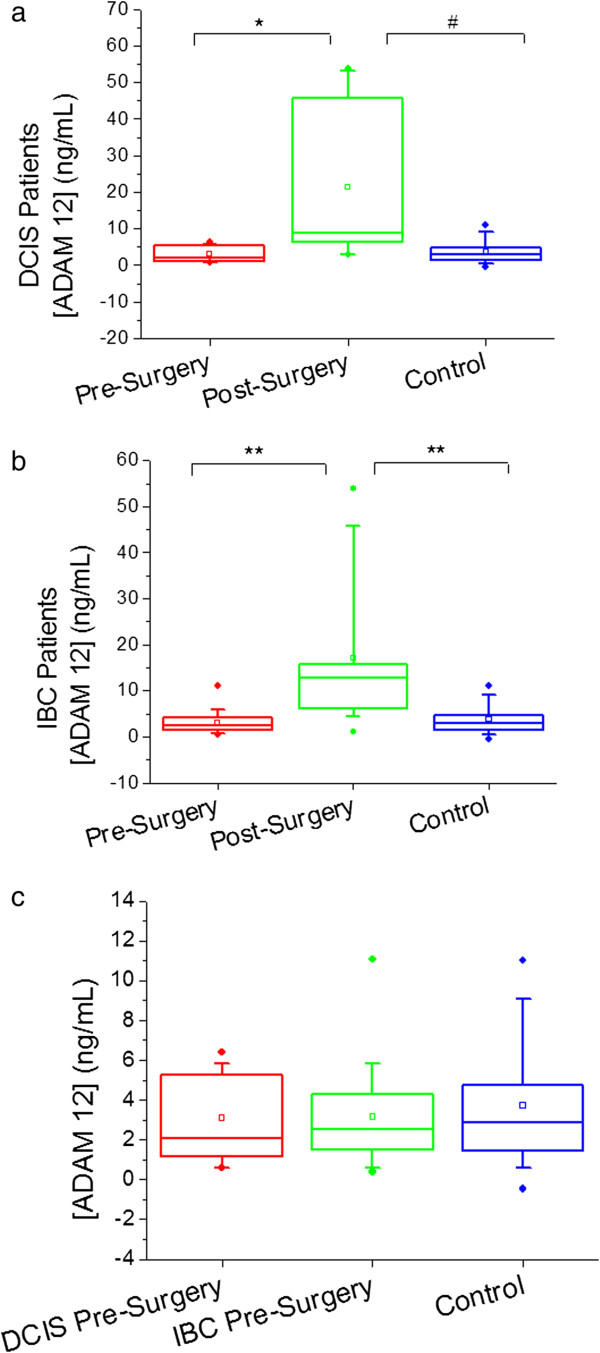
**Boxplots showing urinary ADAM 12 concentration in ng/mL pre- and post- surgery for DCIS patients only (a), IBC patients only (b), and both DCIS and IBC patients pre-surgery (c) with comparison to the control group.** Circles above and below the box denote 99% and 1%, respectively; vertical bars denote 10-90%; the box denotes 25-75%; the small square box denotes the mean; and the horizontal bar denotes the median. * p = 0.0003, # p = 0.0128, ** p < 0.0001.

The cancer patients recruited for this study consist of 15 women with a diagnosis of DCIS, and 35 women with a diagnosis of IBC (30% and 70% of the total group, respectively). If we consider these as separate groups and compare these groups individually to the control group the results do not change, nor do the DCIS and IBC patients differ significantly from each other pre-surgery (see Figure 2). The ADAM 12 concentration ranges and median changes in concentration from pre- to post-surgery are also consistent between the DCIS and IBC groups (see Table 2). Significant elevation of urinary ADAM 12 does take place after patients have undergone surgery (overall p-value < 0.0001).

**Table 2:** Ranges and median change of ADAM 12 concentration for controls and cancer patients (by stage)

**Stage**	**Pre-surgery**	**Post-surgery**	**Median change**
Control	0—11.01 ng/mL	NA	NA
DCIS	0.6—15.8 ng/mL	1.0—53.8 ng/mL	5.4
IBC	0.1—11.1 ng/mL	1.1—53.8 ng/mL	7.5

**Figure 3:**
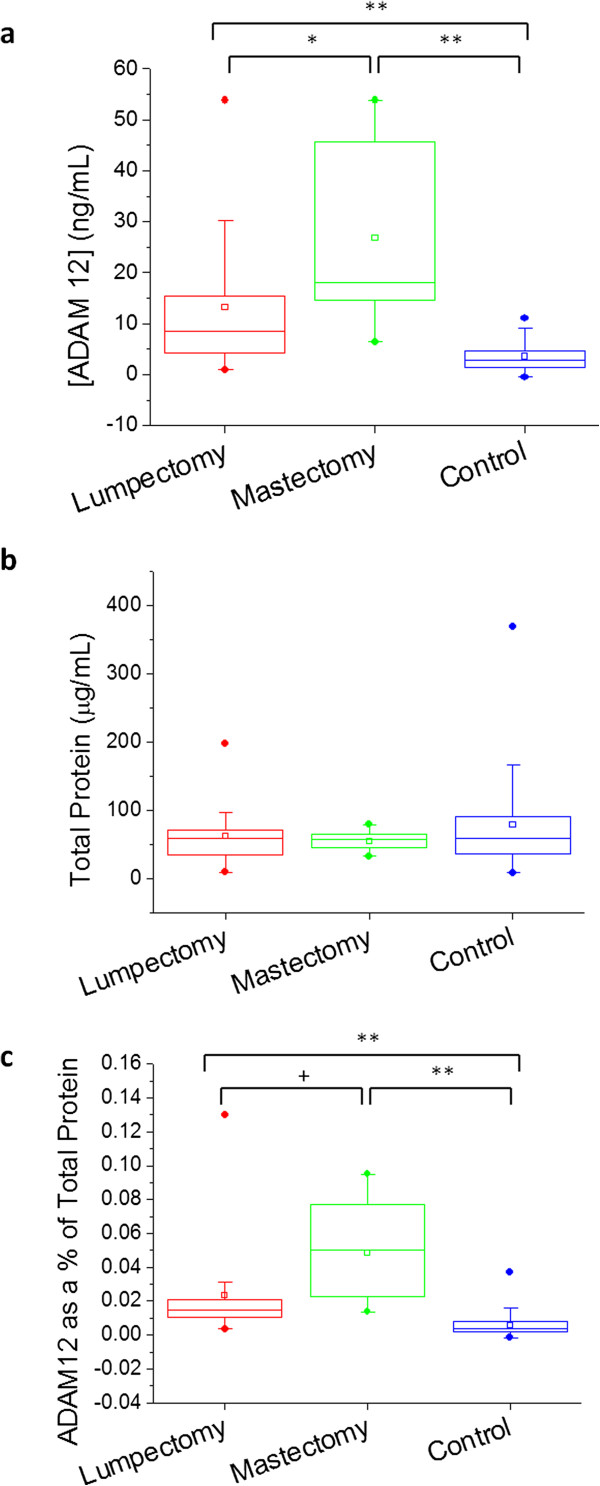
**Boxplots showing urinary ADAM 12 levels in patients who underwent lumpectomies or mastectomies vs. control group; concentration of ADAM 12 denoted in ng/mL (a), total protein concentration (b), and ADAM 12 as a% of total protein (c) are shown for each respective group.** Circles above and below the box denote 99% and 1%, respectively; vertical bars denote 10-90%; the box denotes 25-75%; the small square denotes the mean; and the horizontal bar denotes the median. * p = 0.0075, ** p < 0.0001, + p = 0.0013.

Results further suggest a link between the extent of patient surgery and urinary ADAM 12 elevation. Of the cancer patients recruited for this study, 38 of these underwent lumpectomies, and 11 underwent mastectomies (77.6% and 22.4% of the total group, respectively; one of the recruited patients failed to leave a post-operative sample). The concentration of ADAM 12 in the urine of mastectomy patients was significantly higher than that of lumpectomy patients post-surgery (p = 0.0075); the median increase in ADAM 12 concentration for mastectomy patients was 14.7 ng/mL, versus 4.8 ng/mL for lumpectomy patients. The median percent increase in urinary ADAM 12 concentration following surgery was 313.6% for lumpectomy patients, and 764.5% for mastectomy patients. When considered as a percentage of total protein, the percent urinary ADAM 12 following a mastectomy versus that following a lumpectomy was significant at p = 0.0013. There were no statistically significant differences between the total urine protein concentrations of these groups. These results are summarized in Table 3 and Figure 3. There were no significant differences between the urinary ADAM 12 concentrations of lumpectomy and mastectomy patients prior to their surgeries (data not shown).

**Table 3:** Ranges and median change of ADAM 12 concentration for lumpectomy patients vs. mastectomy patients (compared to controls)

**Surgery**	**Pre-surgery**	**Post-surgery**	**Median change**
Control	0—11.0 ng/mL	NA	NA
Lumpectomy	0.1—15.8 ng/mL	1.0—53.8 ng/mL	4.8
Mastectomy	0.4—7.6 ng/mL	6.4—53.8 ng/mL	14.7

## Discussion

Interestingly, the current results appear to contradict those published in 2004 [11]; while these authors have concluded that patients with DCIS and IBC had significantly higher levels of ADAM 12 in their urine, our data shows no significant difference between the cancer and control groups. It should here be noted that this group has evaluated the patient concentration of urinary ADAM 12 by western blot, and they have used polyclonal antibodies directed against the cysteine rich domain of ADAM 12 in their analysis [11,35]. We believe that this difference in technique alone should not have resulted in these significant variations in results.

In addition, there is also an apparent conflict between the current results and those published in 2012 [39]. These authors have observed an increase in the genetic expression of both ADAM 12 and ADAM 17 in malignant breast tissue. However, it should be noted that their results also suggest that the expression levels of ADAM 12-L (the membrane bound form) are considerably more elevated than those of ADAM 12-S (the secreted form) [39]. With regard to protein expression, Narita, et al. do not distinguish between ADAM 12-L and ADAM 12-S. This suggests that the elevation of ADAM 12 protein seen during this study was likely primarily elevation of ADAM 12-L, and as this form is membrane bound, it is far less likely to end up in the patient’s urine.

The current results raise a number of interesting questions. The observed elevation in ADAM 12 following surgery is not surprising: many matrix metalloproteinase enzymes are upregulated during wound healing [40], and evidence suggests that ADAM 12 is involved in tissue remodeling [41], making it likely to undergo upregulation following surgical or other trauma to the tissues. We also note *a priori* that many patients recruited for this study have co-morbidities which may affect levels of ADAM 12 (e.g. osteoarthritis [32], allergic rhinitis [42], and asthma [43]; however these co-morbidities are well balanced between the cancer group and the control group, and based on our analysis they have had no significant effect on the concentrations of urinary ADAM 12. The data also shows that some cancer and control patients having levels of ADAM 12 above the median did not have obvious co-morbidities. It remains to be determined under what circumstances members of a certain group could have significantly elevated levels of urinary ADAM 12 compared to members of another group, assuming these groups are age-matched. Based on our observations, these circumstances could easily occur if the group having elevated urinary ADAM 12 had undergone surgery within four weeks of having been tested. Further, as those patients with more advanced stages of cancer would be likely to have had more extensive surgery; it would follow that those patients with higher stage breast cancers would appear to have higher urinary ADAM 12 concentrations. Other tissue trauma could also play a role, such as biopsies. Further, if existing co-morbidities had not been well balanced between the control and test groups, it is likely these could have played a role in the elevation of urinary ADAM 12 levels in one group over another.

As a final consideration we note that in 2011, the same group (which concluded in 2004 that DCIS patients have significantly elevated urinary ADAM 12 vs. controls) has conducted another study (utilizing fluorescent metalloproteinase substrates) to simultaneously detect a number of matrix metalloproteinases (MMP-1, -2, -3, -8, -9, and -13) and ADAMs (ADAM-8, -9, -10, -12, and -17) in the urine of cancer patients and age-matched controls. This study concluded that no statistical difference exists between DCIS patients and age-matched controls when this polymer-based method is used [12]. Due to these contradictory results, further studies are necessary in order either to accept or to reject the measurement of urinary ADAM 12 as a viable method for the diagnosis of breast cancer.

## Conclusions

We find no significant difference between urinary ADAM 12 concentrations in patients diagnosed with DCIS or IBC and their age-matched controls prior to any surgery or other therapeutic treatment. Further, we find no significant differences in urinary ADAM 12 concentrations between DCIS patients and IBC patients either prior to or following surgical treatment. These results are in contrast to those published by another group in 2004 [11].

Following surgical treatment, the concentrations of urinary ADAM 12 are elevated significantly over age-matched controls, and the degree of this increase depends upon the extent of the surgery.

These conclusions suggest that an increase in the concentration of urinary ADAM 12 may not correlate directly with the status and stage of breast cancer as previously thought; rather these increases may be a result of tissue injury and inflammation from biopsy and surgical resection. Further studies are necessary to accept or reject the measurement of urinary ADAM 12 as a viable method for the diagnosis of breast cancer.

The above results may suggest a need for biomarkers to be evaluated carefully in the context of tissue damage.

## Abbreviations

ADAM12: A disintegrin and metalloproteinase isoform 12; DCIS: Ductal carcinoma in situ; IBC: Invasive breast cancer; ELISA: Enzyme-linked immunosorbent assay.

## Competing interests

The authors declare that they have no competing interests.

## Authors’ contributions

ENE was involved in study design, carried out ELISA and Bradford assays, compiled data, conducted data analysis and interpretation, and drafted the manuscript. MB assisted in patient recruitment, contributed to experimental design, and assisted in manuscript revision. JT assisted with ELISA procedures, data acquisition, and data interpretation. MR assisted with IRB submission, manuscript review, and study design. SM provided laboratory facilities, assisted with study design and manuscript review. NA conceived of the study, secured the grant for the study, organized facilities for patient recruitment, and approved manuscript drafting and submission. All authors read and approved the final manuscript.
